# From self-care to motivation: health-oriented leadership and basic psychological need satisfaction at work

**DOI:** 10.3389/fpsyg.2026.1807780

**Published:** 2026-06-29

**Authors:** Sofija Kowalski, Johannes Günther von Gehlen

**Affiliations:** 1 Department of Business Psychology, FOM University of Applied Sciences, Nuremberg, Germany; 2 Laboratory of Business Psychology, Department of Business Psychology, Institute of Economic Sciences, Hochschule Neu-Ulm, Neu-Ulm, Germany

**Keywords:** basic psychological need satisfaction, employee self-care, health-oriented leadership, intrinsic motivation, leader self-care, occupational health psychology, self-determination theory, staff-care

## Abstract

Leadership research increasingly emphasizes the role of health-oriented leadership (HoL) for employee well-being and motivation, yet the motivational relevance of leaders’ own self-care remains insufficiently understood. Addressing this gap, the present study integrates the HoL framework with self-determination theory (SDT) to examine how leader self-care relates to employees’ basic psychological need satisfaction and intrinsic motivation. Building on SDT, self-care is conceptualized as a form of health-related self-regulation that supports the satisfaction of the basic psychological needs for autonomy, competence, and relatedness. The study proposes a theoretically ordered model in which leader self-care is associated with staff-care, employee self-care, need satisfaction, and intrinsic motivation, thereby extending prior research that has primarily focused on leaders’ health-supportive behavior toward employees. Data were collected from 122 employees in Germany using a cross-sectional online survey. Regression analyses showed that leader self-care was a strong and significant predictor of staff-care (*b* = 1.01, *p* < 0.001) and employee self-care (*b* = 0.37, *p* < 0.001). Within the mediation model, staff-care was positively but not significantly associated with basic psychological need satisfaction (*b* = 0.12, *p* = 0.055), and the indirect effect of leader self-care on need satisfaction via staff-care was not statistically significant (*b* = 0.12, 95% CI [0.00, 0.25]). In contrast, employee self-care was a significant predictor of need satisfaction (*b* = 0.41, *p* < 0.001), which in turn strongly predicted intrinsic motivation (*b* = 1.06, *p* < 0.001). These findings suggest that HoL may influence motivation less through direct supportive behavior and more through enabling employees’ self-regulatory engagement. By identifying employee self-care as a proximal correlate of need satisfaction, the study contributes to a more differentiated understanding of how leadership-related health resources translate into motivational outcomes. The study is limited by its cross-sectional design, reliance on self-report data, and geographically restricted sample. Future research should employ longitudinal and multi-source designs to examine causal mechanisms and contextual boundary conditions.

## 1 Introduction

Psychological sustainability in contemporary work environments increasingly depends on leadership practices that support employee well-being and motivation. In light of rising labor market mobility, demographic change, and increasing work demands, organizations face growing challenges in maintaining a healthy and motivated workforce (Hammermann et al., 2022; Krist and Baumgardt, 2024). A substantial body of research shows that leaders play a central role in shaping employees’ psychological functioning by influencing interpersonal climates and work conditions (Montaño et al., 2016; Wegge et al., 2014). At the same time, organizational research emphasizes that employee health and well-being are not solely individual responsibilities but are fundamentally shaped by structural and social working conditions (Van Dick, 2023). Consequently, effective workplace health management requires the integration of leadership behavior with broader organizational systems (Uhle and Treier, 2019).

Despite this growing consensus, a critical question remains insufficiently understood: how do leaders’ own health-related behaviors contribute to motivational processes at work? Existing research on health-oriented leadership (HoL) has primarily focused on leaders’ supportive behavior toward employees, demonstrating its relevance for employee well-being and mental health. However, the role of leader self-care as a potential upstream resource has received comparatively little attention. At the same time, motivational research—particularly self-determination theory (SDT; Deci and Ryan, 2000)—has extensively explained how intrinsic motivation emerges through the satisfaction of the basic psychological needs for autonomy, competence, and relatedness. Yet, these two perspectives have rarely been systematically integrated.

This lack of integration represents a central theoretical gap. While leadership research focuses on whether leaders support employee health, SDT explains why employees become motivated. What remains unclear is how leadership-related health behavior translates into motivational outcomes. In particular, it is not well understood whether leadership directly influences motivation or whether its effects depend on employees’ own self-regulatory engagement.

The present study addresses this gap by integrating the HoL framework (Franke et al., 2015; Pundt and Felfe, 2017) with SDT. HoL distinguishes between leader self-care, staff-care, and employee self-care and thus provides a differentiated perspective on health-related leadership processes. Building on this framework, the present study examines whether employee self-care represents a proximal mechanism through which leadership-related resources relate to basic psychological need satisfaction and intrinsic motivation.

By doing so, the study moves beyond a direct leadership-effects perspective and proposes a more differentiated mechanism in which leadership shapes motivational outcomes indirectly through employees’ self-regulatory processes. Specifically, it examines whether leader self-care is associated with staff-care and employee self-care, and whether these variables are differentially related to basic psychological need satisfaction and intrinsic motivation.

The study makes three contributions. First, it contributes theoretically by integrating HoL with SDT and by conceptualizing self-care as a health-related self-regulatory resource within motivational processes. Second, it contributes empirically by differentiating between distal leadership-related resources (staff-care) and proximal self-regulatory mechanisms (employee self-care). Third, it contributes practically by identifying self-care and staff-care as trainable competencies that may be relevant for leadership development and organizational health promotion.

## 2 Theoretical background

### 2.1 Intrinsic motivation

Motivation research has consistently demonstrated that human behavior cannot be explained solely by external rewards or punishment. Early studies showed that individuals may engage in activities out of intrinsic interest (Harlow, 1950), and subsequent research highlighted that external rewards can even undermine intrinsic motivation when perceived as controlling (Deci, 1971; Lepper and Greene, 1975; Pritchard et al., 1977).

Intrinsic motivation refers to engaging in an activity for its inherent satisfaction rather than for external outcomes. Within SDT (Deci and Ryan, 2000), intrinsic motivation represents a high-quality form of motivation that emerges when individuals experience their work as meaningful, self-endorsed, and personally engaging. In organizational contexts, intrinsic motivation is particularly relevant because it is associated with engagement, persistence, performance, creativity, and well-being (Gagné et al., 2015; Grünwald et al., 2024).

Importantly, SDT emphasizes that intrinsic motivation is shaped by social and contextual conditions. Leadership may therefore play an important role in creating work environments that support motivational quality.

### 2.2 Basic psychological need satisfaction

According to SDT, optimal functioning and intrinsic motivation depend on the satisfaction of three basic psychological needs: autonomy, competence, and relatedness (Deci and Ryan, 2000). Autonomy refers to acting in accordance with one’s values, competence reflects the experience of effectiveness, and relatedness captures the need for social connection. These needs have been shown to operate as universal psychological mechanisms across contexts and cultures (Chen et al., 2014; Hahn and Oishi, 2006).

Within organizational settings, basic psychological need satisfaction represents a central mechanism through which contextual conditions are translated into motivational outcomes. Employees are more likely to experience intrinsic motivation when they feel autonomous in their work, competent in handling tasks, and socially connected to others. Empirical research has consistently linked need satisfaction to intrinsic motivation and work-related outcomes such as engagement, performance, and well-being (Gagné et al., 2015; Grünwald et al., 2024).

Consistent with serial mediation findings, basic psychological need satisfaction should be understood as a central motivational mechanism rather than a mere outcome variable, as it can mediate the relationship between leadership behavior and intrinsic motivation across organizational contexts (Hudecek et al., 2024). This is particularly relevant for the present study, as basic psychological need satisfaction is positioned as the key motivational mechanism linking health-related leadership resources and employee self-regulation to intrinsic motivation.

### 2.3 Leadership and health-oriented leadership

Leadership represents a central contextual factor influencing both employee health and motivation. Research shows that leaders shape employees’ experiences through their behavior, the work conditions they create, and their role-modeling function (Elprana et al., 2016; Krick et al., 2021). While leadership research has traditionally focused on performance and motivation, more recent approaches highlight its relevance for employee well-being and health.

The HoL framework (Franke et al., 2015; Pundt and Felfe, 2017) provides a differentiated perspective on health-related leadership processes. It distinguishes between leader self-care, staff-care, and employee self-care. Leader self-care refers to leaders’ awareness of and behavior toward their own health, staff-care captures health-supportive leadership behavior directed toward employees, and employee self-care reflects individuals’ own health-related self-regulation.

A central assumption of HoL is that leader self-care represents a foundational resource that shapes both leadership behavior and employee outcomes. Leaders who actively engage in self-care are more likely to demonstrate staff-care and may encourage employee self-care through role-modeling processes (Franke et al., 2015; Klug et al., 2022). Empirical research supports these assumptions, showing that HoL and consistent self-care and staff-care profiles are associated with employee well-being, reduced strain, and improved health outcomes (Klug et al., 2019; Klebe et al., 2021; Stuber et al., 2020; Vonderlin et al., 2021).

However, prior research has predominantly focused on health-related outcomes, while the relationship between HoL and motivational processes remains less well understood.

### 2.4 Self-care as a self-regulatory resource

Self-care can be conceptualized as a form of health-related self-regulation aimed at maintaining and restoring psychological and physical resources. In organizational contexts, self-care includes behaviors such as recovery, stress management, and boundary regulation, which enable individuals to cope with job demands and sustain performance.

Within the HoL framework, self-care operates at both the leader and employee level. While leader self-care shapes leadership behavior, employee self-care represents a more proximal mechanism through which individuals actively regulate their resources. Importantly, self-care is not merely a health-related construct but may also be relevant for motivational processes, as it enables individuals to maintain the psychological conditions necessary for experiencing autonomy, competence, and relatedness.

From this perspective, employee self-care may help explain why health-related leadership resources do not automatically translate into motivation. Instead, employees may need to actively use and enact available resources in order to experience need satisfaction and intrinsic motivation.

### 2.5 Integrating health-oriented leadership and self-determination theory

The present study integrates HoL with SDT to explain how HoL relates to employee motivation. While HoL focuses on health-related regulation processes, SDT explains how these processes translate into motivational outcomes.

From this perspective, leader self-care can be conceptualized as an upstream resource that shapes staff-care and employee self-care. Staff-care may provide a supportive contextual environment, whereas employee self-care represents a proximal self-regulatory mechanism through which individuals actively maintain resources and experience need satisfaction.

Thus, basic psychological need satisfaction functions as the central mechanism linking leadership-related resources to intrinsic motivation. This integrated perspective suggests that leadership may not directly produce motivation but rather creates conditions under which employees engage in self-regulatory processes that foster motivation.

Despite these theoretical links, empirical research integrating HoL and SDT remains scarce. In particular, the role of employee self-care as a proximal mechanism within SDT-based motivational processes has not been systematically examined. The present study addresses this gap by proposing that leadership-related health regulation influences motivation not only through direct supportive behavior but also through employees’ active self-regulation.

## 3 Hypotheses

Based on the HoL framework and its integration with SDT, the following hypotheses are proposed.

Leader self-care is conceptualized as a foundational resource that shapes leadership behavior. Leaders who actively regulate their own health are expected to demonstrate higher levels of staff-care.

*H1*: Leader self-care is positively associated with staff-care.

Health-supportive leadership may create working conditions that foster autonomy, competence, and relatedness. Therefore, staff-care is expected to be positively related to basic psychological need satisfaction.

*H2*: Staff-care is positively associated with basic psychological need satisfaction.

Leader self-care may also influence employees’ own health-related behavior through role-modeling processes.

*H3*: Leader self-care is positively associated with employee self-care.

Employee self-care represents a proximal self-regulatory mechanism and is expected to be directly related to need satisfaction.

*H4*: Employee self-care is positively associated with basic psychological need satisfaction.

Consistent with SDT, need satisfaction is expected to predict intrinsic motivation.

*H5*: Basic psychological need satisfaction is positively associated with intrinsic motivation.

**Figure 1:**
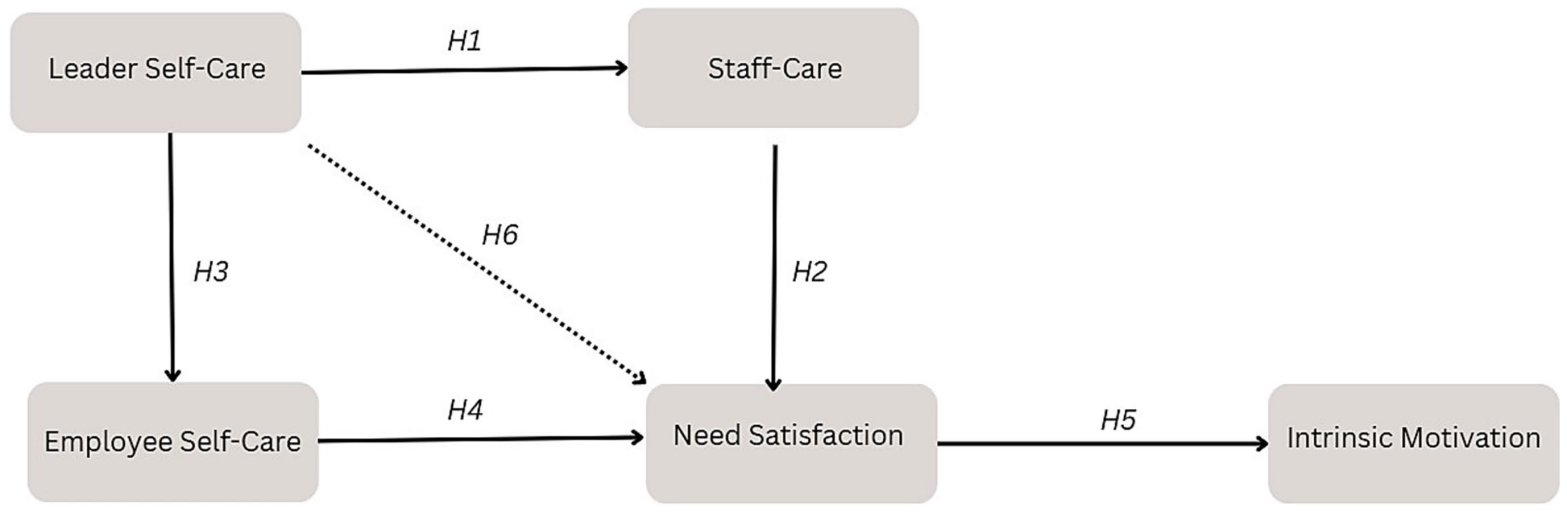
Summarizes the hypothesized model.

Finally, staff-care may function as a contextual pathway linking leader self-care to need satisfaction (Figure 1).

*H6*: Staff-care mediates the relationship between leader self-care and basic psychological need satisfaction.

## 4 Materials and methods

### 4.1 Study design and procedure

A quantitative, non-experimental cross-sectional design was employed using an online survey administered via SoSci Survey. Data were collected in Germany between September 3 and October 31, 2024. Participants were recruited through social media and professional networks.

Eligibility criteria required participants to be employed under direct supervision without holding managerial responsibilities. Participation was voluntary and anonymous, and informed consent was obtained from all respondents. The study adhered to institutional and national ethical standards.

A pretest (*N* = 5) was conducted to assess clarity, survey duration, and technical functionality, resulting in minor wording adjustments. Attention-check items were included to ensure data quality, and responses were screened prior to analysis.

### 4.2 *A priori* power analysis

An *a priori* power analysis was conducted using G*Power 3.1. Assuming a medium effect size (*f*^2^ = 0.15), *α* = 0.05, and statistical power of 0.80, a minimum sample size of *N* = 92 was required for regression analyses with up to three predictors.

For mediation analyses, recommended sample sizes for detecting small-to-medium indirect effects range between 100 and 120 (Fritz and MacKinnon, 2007). The final sample (*N* = 122) exceeded these thresholds, indicating sufficient statistical power.

### 4.3 Participants

A total of 126 individuals completed the survey. Four cases were excluded due to failed attention checks (*n* = 2) or straight-lining (*n* = 2), resulting in a final sample of *N* = 122.

Participants were 61.5% female, 37.7% male, and 0.8% diverse. Age ranged from 19 to 57 years (M = 33.23, SD = 8.86), and mean job tenure was 7.24 years (SD = 5.71). Participants represented diverse sectors, including healthcare, administration, and education.

### 4.4 Measures

#### 4.4.1 Health-oriented leadership

Leader self-care, staff-care, and employee self-care were assessed using the validated HoL follower questionnaire (Pundt and Felfe, 2017). The scales comprised 14 items (leader self-care), 31 items (staff-care), and 19 items (employee self-care), rated on five-point Likert scales (1 = strongly disagree to 5 = strongly agree).

Staff-care reflects perceived health-supportive leadership behavior rather than objective behavior. Subscales assessed health awareness, valuation of health, and health behavior. Internal consistencies ranged from *α* = 0.67 to 0.87.

#### 4.4.2 Basic psychological need satisfaction

Need satisfaction was measured using the W-BNS-G (Grünwald et al., 2024), assessing autonomy, competence, and relatedness on a five-point Likert scale. Internal consistencies ranged from α = 0.77 to 0.83.

#### 4.4.3 Intrinsic motivation

Intrinsic motivation was assessed using the three-item subscale of the Multidimensional Work Motivation Scale (Gagné et al., 2015), rated on a seven-point scale. Reliability was high (α = 0.85).

### 4.5 Data preparation

All items were mandatory, resulting in no missing data. Negatively worded items were reverse-coded, and scale scores were computed as mean values. Assumptions for regression analyses were checked prior to hypothesis testing.

### 4.6 Statistical analysis

Analyses were conducted using R (version 4.3.2; R Core Team, 2024). Descriptive statistics and Pearson correlations were calculated to examine variable distributions and bivariate relationships.

Hypotheses H1, H3, H4, and H5 were tested using linear regression analyses. Hypothesis H2 was examined as the b-path within the mediation model, and Hypothesis H6 was tested using bootstrapped mediation analysis with 10,000 resamples based on Hayes (2018).

Indirect effects were considered significant if 95% confidence intervals did not include zero (Zhao et al., 2010). Continuous predictors were mean-centered. Effect sizes were reported as standardized coefficients (*β*) and *R*^2^ values.

Model fit indices (RMSEA, CFI, TLI, SRMR) were reported; however, as the model was fully saturated (*df* = 0), fit indices were not interpreted substantively.

## 5 Results

### 5.1 Descriptive statistics

Descriptive statistics indicated that leader self-care (M = 3.31, SD = 0.70) and employee self-care (M = 3.53, SD = 0.64) were above the scale midpoint, suggesting relatively high levels of health-oriented behavior in the sample. Staff-care (M = 3.24, SD = 0.89) and basic psychological need satisfaction (M = 3.33, SD = 0.47) were moderately pronounced. Intrinsic motivation showed a comparatively higher mean level (M = 5.29, SD = 1.44).

As shown in Table 1, Pearson correlations revealed substantial positive associations among the core variables. Leader self-care was strongly correlated with staff-care (*r* = 0.79, *p* < 0.01) and employee self-care (*r* = 0.76, *p* < 0.01). Staff-care was also strongly associated with employee self-care (*r* = 0.82, *p* < 0.01). Basic psychological need satisfaction was positively correlated with leader self-care (*r* = 0.51, *p* < 0.01), staff-care (*r* = 0.49, *p* < 0.01), and employee self-care (*r* = 0.55, *p* < 0.01). Intrinsic motivation was positively correlated with leader self-care (*r* = 0.36, *p* < 0.01), staff-care (*r* = 0.32, *p* < 0.01), employee self-care (*r* = 0.30, *p* < 0.01), and basic psychological need satisfaction (*r* = 0.35, *p* < 0.01).

**Table 1:** Bivariate correlations among study variables.

Variable	1	2	3	4	5
1. Leader self-care	—				
2. Staff-care	0.79**	—			
3. Employee self-care	0.76**	0.82**	—		
4. Basic psychological need satisfaction	0.51**	0.49**	0.55**	—	
5. Intrinsic motivation	0.36**	0.32**	0.30**	0.35**	—

*N* = 122. Values represent Pearson correlations. **p < 0.01. Leader self-care, staff-care, and employee self-care were measured on 5-point Likert scales (1 = strongly disagree to 5 = strongly agree). Basic psychological need satisfaction was measured on a 5-point scale (1 = not at all true to 5 = completely true). Intrinsic motivation was measured on a 7-point scale (1 = not at all to 7 = very strongly).

Distributional assumptions were examined using Shapiro–Wilk tests and visual inspection of histograms and Q–Q plots. Shapiro–Wilk tests indicated significant deviations from normality for staff-care (W = 0.92, *p* = 2.11e-06), leader self-care (W = 0.96, *p* = 0.0005), employee self-care (W = 0.95, *p* = 0.0003), basic psychological need satisfaction (W = 0.94, *p* = 5.81e-05), and intrinsic motivation (W = 0.89, *p* = 7.46e-08). However, visual inspection of histograms and Q–Q plots did not indicate severe distributional problems. Given the sample size, the robustness of regression-based procedures, and the use of bootstrapped confidence intervals for the mediation analysis, these deviations were not considered critical for the interpretation of the results.

To assess potential multicollinearity, variance inflation factors (VIFs) were calculated. Values ranged between 1.62 and 2.68, indicating no evidence of problematic multicollinearity.

### 5.2 Hypothesis testing

Hypotheses were tested using linear regression analyses and bootstrapped mediation procedures (10,000 resamples; *α* = 0.05). Standardized regression coefficients (*β*), unstandardized coefficients (b), and explained variance (*R*^2^) are reported.

#### 5.2.1 H1: leader self-care → staff-care

To test Hypothesis 1, a linear regression analysis was conducted with leader self-care as the predictor of staff-care. The results revealed a strong and statistically significant positive effect (*b* = 1.01, SE = 0.05, *β* = 0.79, *p* < 0.001). The model explained a substantial proportion of variance (*R*^2^ = 0.63), indicating that leader self-care accounts for 63% of the variability in staff-care. Following Cohen (1988), this effect can be interpreted as large.

Hypothesis 1 was supported.

#### 5.2.2 H2: staff-care → need satisfaction

Hypothesis 2 was examined within the mediation model as the *b-path* linking staff-care to basic psychological need satisfaction. The results indicated a small-to-medium positive association that did not reach conventional levels of statistical significance (*b* = 0.12, SE = 0.06, *β* = 0.23, *p* = 0.055, 95% CI [0.00, 0.24]). Following Cohen (1988), the effect size of the b-path can be interpreted as small to medium.

Although staff-care was positively correlated with basic psychological need satisfaction (*r* = 0.49, *p* < 0.01; see Table 1), the effect was not statistically significant within the mediation model. This discrepancy may be explained by shared variance with leader self-care, which reduced the unique contribution of staff-care within the mediation model.

Accordingly, Hypothesis 2 was not supported.

#### 5.2.3 H3: leader self-care → employee self-care

A regression analysis was conducted to examine the relationship between leader self-care and employee self-care. The results indicated a strong and statistically significant positive effect (*b* = 0.37, SE = 0.03, *β* = 0.76, *p* < 0.001). The model explained 58% of the variance in employee self-care (*R*^2^ = 0.58). Following Cohen (1988), this effect can be interpreted as large.

Hypothesis 3 was supported.

#### 5.2.4 H4: employee self-care → need satisfaction

To test Hypothesis 4, employee self-care was regressed on basic psychological need satisfaction. The analysis revealed a significant positive effect (*b* = 0.41, SE = 0.07, *β* = 0.46, *p* < 0.001), explaining 21% of the variance (*R*^2^ = 0.21). Following Cohen (1988), this effect can be interpreted as medium in size.

Compared to staff-care, employee self-care accounted for a substantially larger proportion of variance in need satisfaction, indicating that individual self-regulatory processes may represent more proximal determinants of motivational experiences.

Hypothesis 4 was supported.

#### 5.2.5 H5: need satisfaction → intrinsic motivation

Hypothesis 5 was examined using linear regression. Basic psychological need satisfaction emerged as a strong and statistically significant predictor of intrinsic motivation (*b* = 1.06, SE = 0.14, *β* = 0.63, *p* < 0.001), explaining 40% of the variance (*R*^2^ = 0.40). Following Cohen (1988), this effect can be interpreted as large.

Hypothesis 5 was supported.

#### 5.2.6 H6: mediation via staff-care

Hypothesis 6 was tested using bootstrapped mediation analysis. The indirect effect of leader self-care on need satisfaction via staff-care was small and not statistically significant (indirect effect = 0.12, SE = 0.06, *β* = 0.18, 95% CI [0.00, 0.25]), as the confidence interval included zero.

The total effect of leader self-care on need satisfaction was significant (*b* = 0.35, SE = 0.08, *β* = 0.46, *p* < 0.001). The direct effect remained significant after controlling for staff-care (*b* = 0.22, SE = 0.10, *β* = 0.29, *p* = 0.025), indicating that the relationship is not primarily transmitted through staff-care.

Hypothesis 6 was not supported.

### 5.3 Model fit

Model fit was evaluated using standard fit indices (RMSEA, CFI, TLI, SRMR). As the mediation model was fully saturated (*df* = 0), all indices indicated perfect fit (RMSEA = 0, CFI = 1, TLI = 1, SRMR = 0). These values reflect the mathematical structure of the model and are not informative for evaluating model adequacy.

### 5.4 Summary of findings

Overall, four of the six hypotheses were supported. Leader self-care emerged as a strong and consistent predictor of both staff-care and employee self-care. Employee self-care was significantly associated with basic psychological need satisfaction, which in turn strongly predicted intrinsic motivation.

In contrast, staff-care was not significantly related to need satisfaction, and the hypothesized indirect effect via staff-care was not supported. The overall pattern of results suggests a differentiation between distal leadership-related influences (staff-care) and more proximal self-regulatory mechanisms (employee self-care) in explaining motivational outcomes.

Importantly, the results should be interpreted as theoretically ordered associations rather than evidence of causal effects due to the cross-sectional design.

## 6 Discussion

### 6.1 Summary of findings

The present study examined how HoL relates to employee motivation by integrating the HoL framework with SDT (Deci and Ryan, 2000; Franke et al., 2015; Pundt and Felfe, 2017). The findings reveal a differentiated pattern in which leadership is associated with motivation primarily through employees’ self-regulatory processes rather than through direct supportive behavior alone.

Leader self-care emerged as a strong and consistent predictor of both staff-care and employee self-care, supporting the assumption that leaders’ own health-related practices shape both leadership behavior and employees’ self-regulatory engagement (Franke et al., 2015; Klug et al., 2022). In contrast, staff-care was not significantly associated with basic psychological need satisfaction. Instead, employee self-care showed a robust positive relationship with need satisfaction, which in turn strongly predicted intrinsic motivation, consistent with SDT (Deci and Ryan, 2000; Gagné et al., 2015).

Taken together, these findings suggest that leadership may not directly generate motivational states but rather shapes the conditions under which employees actively engage in self-regulatory processes that foster motivation. This aligns with broader perspectives emphasizing that work motivation emerges from the interaction between contextual conditions and individual self-regulation (Hackman and Oldham, 1976; Van Dick, 2023).

### 6.2 Relation to prior research

The results are consistent with SDT in demonstrating that basic psychological need satisfaction represents a central predictor of intrinsic motivation (Deci and Ryan, 2000; Gagné et al., 2015; Chen et al., 2014). This finding is consistent with evidence from two independent samples (*N* = 1,306) demonstrating that basic psychological need satisfaction robustly mediates the relationship between leadership behavior and intrinsic motivation across different organizational contexts (Hudecek et al., 2024). At the same time, they extend prior leadership research by suggesting that the motivational effects of health-supportive leadership are more indirect than previously assumed. While prior studies have demonstrated that leadership styles such as servant leadership are positively associated with employees’ motivational quality (Hudecek et al., 2024), the present findings indicate that these effects may operate through more indirect, self-regulatory mechanisms.

Previous studies have primarily linked staff-care to employee well-being, reduced strain, and mental health outcomes (Vonderlin et al., 2021; Klebe et al., 2021). In contrast, the present findings indicate that its association with motivational outcomes is comparatively weak when considered in isolation. This challenges the assumption that supportive leadership behavior directly translates into motivational experiences.

This interpretation is consistent with research emphasizing that leadership provides resources, whereas outcomes depend on how employees utilize these resources (Kohnen et al., 2024). Similarly, research on workplace climates suggests that contextual support becomes effective only when employees actively engage with it (Legault et al., 2025). This interpretation is further supported by findings showing that compassion experienced from others is more strongly related to secure flourishing and organizational commitment than compassion enacted toward others, suggesting that supportive social resources become most relevant through employees’ subjective experience of support (Ford et al., 2024).

Moreover, the findings align with social learning and role-modeling perspectives (Bandura, 1977), suggesting that leadership effects operate less through direct behavioral influence and more through enabling and legitimizing employees’ own self-regulatory behavior (Krick et al., 2021; Klug et al., 2022). In this sense, leader self-care may function as a behavioral signal that encourages employees to adopt similar health-related practices.

### 6.3 Theoretical implications

The present study contributes to leadership and motivation research in several important ways.

First, it extends SDT by introducing employee self-care as a relevant self-regulatory mechanism within motivational processes. While SDT emphasizes the role of contextual support in fostering need satisfaction (Deci and Ryan, 2000), the present findings suggest that individual self-regulation represents a more proximal determinant of need satisfaction than leadership behavior alone. This complements SDT by highlighting the active role of employees in translating contextual resources into motivational experiences.

Second, the study refines the HoL framework (Franke et al., 2015; Pundt and Felfe, 2017) by differentiating between distal and proximal mechanisms. Staff-care appears to function primarily as a contextual facilitator, whereas employee self-care represents a proximal enactment mechanism directly linked to daily experiences of autonomy, competence, and relatedness. This distinction aligns with multilevel perspectives in organizational psychology that differentiate between contextual resources and individual resource utilization (Van Dick, 2023).

Third, the findings position leader self-care as an upstream resource that shapes both leadership behavior and employee self-regulation. This supports the assumption that leadership effects are partly transmitted through role-modeling processes (Franke et al., 2015; Klug et al., 2022) and highlights the importance of leaders’ own health behavior as a foundational component of effective leadership.

At the same time, the high correlations among HoL dimensions raise questions regarding conceptual distinctiveness. It remains unclear to what extent leader self-care, staff-care, and employee self-care represent distinct constructs or reflect broader leadership perceptions. Future research should therefore examine their discriminant validity in relation to established leadership constructs such as transformational leadership (Bass, 1990), servant leadership (van Dierendonck, 2011), or Leader–Member Exchange (Graen and Uhl-Bien, 1995).

### 6.4 Practical implications

The findings have several implications for leadership development and organizational health management.

First, leadership development programs should explicitly address leader self-care as a foundational competency. Leaders who actively regulate their own health appear more likely to engage in health-supportive leadership behavior and to foster similar behaviors among employees (Krick and Felfe, 2024).

Second, interventions focusing solely on staff-care may be insufficient to influence motivation. Organizations should therefore combine leadership training with structures that support employee self-regulation, such as recovery opportunities, boundary management practices, and autonomy-supportive work design (Hasselmann, 2017; Freudenberg, 2024). Participatory and HR-led intervention approaches may be particularly effective in translating these structures into practice, as they actively involve employees in the development and implementation of well-being initiatives and thereby strengthen self-regulatory engagement (Shekhar et al., 2025).

Third, the findings highlight the importance of combining contextual and individual-level approaches. Leadership can create enabling conditions, but sustainable motivation depends on employees’ ability to translate these conditions into self-regulatory action. This aligns with organizational health management approaches that emphasize the integration of structural, leadership-related, and individual-level interventions (Uhle and Treier, 2019).

### 6.5 Limitations and future research

Several limitations should be considered when interpreting the findings.

First, the cross-sectional design does not allow causal conclusions. Although the theoretical model assumes directional relationships, longitudinal, diary-based, and experimental designs are needed to examine causal and temporal dynamics more precisely.

Second, the exclusive reliance on self-report data increases the risk of common method bias. While self-reports are appropriate for capturing subjective perceptions, future research should incorporate multi-source data, including leader ratings, peer evaluations, and objective organizational indicators.

Third, the sample was restricted to employees in Germany, limiting generalizability to other cultural and organizational contexts. Future studies should examine whether the observed relationships hold across different countries and industries.

Fourth, the high correlations among HoL dimensions suggest potential conceptual overlap. Future research should further investigate whether these constructs represent distinct processes or broader evaluative tendencies toward leadership and the work environment.

Finally, the present study did not examine boundary conditions. Future research should investigate moderating factors such as job autonomy, workload, organizational climate, and employees’ baseline well-being to better understand when leadership-related resources translate into motivational outcomes (Hackman and Oldham, 1976; Van Dick, 2023). This is particularly relevant because recent research suggests that the effectiveness of mindfulness- and self-regulation-related resources may depend on employees’ baseline well-being (Monzani et al., 2024).

## 7 Conclusion

The present study demonstrates that HoL may influence employee motivation not primarily through direct supportive behavior, but through enabling employees’ self-regulatory engagement.

By integrating the HoL framework with SDT, the findings identify employee self-care as a central mechanism linking leadership-related resources to intrinsic motivation. Leadership thus appears to function less as a direct driver of motivation and more as a context-shaping force that enables employees to actively regulate their own psychological and health-related resources.

Overall, the results suggest that sustainable employee motivation depends on the interaction between contextual support and individual self-regulatory processes, highlighting the importance of combining leadership development with structures that support employee self-care.

## Data Availability

The raw data supporting the conclusions of this article will be made available by the authors, without undue reservation.
